# Cathode lens spectromicroscopy: methodology and applications

**DOI:** 10.3762/bjnano.5.198

**Published:** 2014-10-27

**Authors:** T O Menteş, G Zamborlini, A Sala, A Locatelli

**Affiliations:** 1 Elettra - Sincrotrone Trieste S.C.p.A., Basovizza, Trieste 34149, Italy; 2 Department of Physics, University of Trieste, Via Valerio 2, Trieste 34137, Italy; 3 Peter Grünberg Institute (PGI-6) and JARA-FIT, Research Center Jülich, 52425 Jülich, Germany

**Keywords:** gold (Au), graphene, intercalation, low-energy electron microscopy (LEEM), magnetism, nanostructures, X-ray magnetic circular dichroism (XMCD), X-ray photoemission electron microscopy (XPEEM)

## Abstract

The implementation of imaging techniques with low-energy electrons at synchrotron laboratories allowed for significant advancement in the field of spectromicroscopy. The spectroscopic photoemission and low energy electron microscope, SPELEEM, is a notable example. We summarize the multitechnique capabilities of the SPELEEM instrument, reporting on the instrumental aspects and the latest developments on the technical side. We briefly review applications, which are grouped into two main scientific fields. The first one covers different aspects of graphene physics. In particular, we highlight the recent work on graphene/Ir(100). Here, SPELEEM was employed to monitor the changes in the electronic structure that occur for different film morphologies and during the intercalation of Au. The Au monolayer, which creeps under graphene from the film edges, efficiently decouples the graphene from the substrate lowering the Dirac energy from 0.42 eV to 0.1 eV. The second field combines magnetism studies at the mesoscopic length scale with self-organized systems featuring ordered nanostructures. This example highlights the possibility to monitor growth processes in real time and combine chemical characterization with X-ray magnetic circular dichroism–photoemission electron microscopy (XMCD–PEEM) magnetic imaging by using the variable photon polarization and energy available at the synchrotron source.

## Introduction

The cathode lens, or immersion objective lens, is used to image electrons emitted from surfaces [1]. In a microscope that uses this type of objective, the sample surface acts as the cathode held at a negative potential, whereas the anode (objective lens) has a central aperture to allow for the passage of the emitted electrons towards the imaging column. The imaged electrons may originate from different processes such as thermionic emission, secondary emission, emission of photoelectrons from core levels and the valence band or elastic backscattering [2]. Methods based on the latter two, photoemission electron microscopy (PEEM) and low energy electron microscopy (LEEM), have found a special place in the field of surface science, and they will be the focus of our review.

During the evolution of PEEM [3], its first use as an X-ray microscope in a synchrotron environment in the late eighties stands out as one of the most significant developments [4]. Since then X-ray PEEM (XPEEM) has become a widespread analytical technique for surface investigation, which takes advantage of the high photon flux along with the tunable energy and polarization available at synchrotron sources [5]. In recent years, the natural combination of XPEEM with LEEM has created the powerful surface science facility, spectroscopic photoemission and low energy electron microscope (SPELEEM). In SPELEEM, the structural sensitivity of LEEM perfectly complements the chemical and magnetic information provided by XPEEM, thus creating a complete characterization tool of material properties at the nanometer length scale.

The following provides an overview on SPELEEM methods along with the recent examples predominantly considering the activity carried out at the Elettra Sincrotrone Trieste. The first part of the paper is organized as an extended introduction to LEEM and XPEEM methodology. Then, we give a detailed account of the instrumental aspects specific to the SPELEEM instrument at Elettra.

The bulk of this work is dedicated to applications of the SPELEEM technique. We put special emphasis on graphene, which has been extensively studied by using cathode lens microscopy, LEEM in particular, with numerous studies of epitaxial graphene grown on a variety of transition metal and silicon carbide supports. These microscopy experiments have been carried out by using well-established methodologies, which were formerly developed for the analysis of ultra-thin metal films on single crystal surfaces [6]. These methods were soon adapted to the needs of the rising research field of graphene, making LEEM one of the prominent methods to access the structural properties of graphene [7]. Since several review works have already addressed this subject [8–10], treating in depth also the experimental methods, the section on graphene is limited to an overview on the most recent research activity. The versatility of the LEEM and SPELEEM methodologies will be further illustrated by the effect of Au intercalation in graphene on Ir(100). The last part of the paper focuses on the studies of magnetism at the nanoscale using the SPELEEM.

## Review

### Low energy electron microscopy

**Figure 1:**
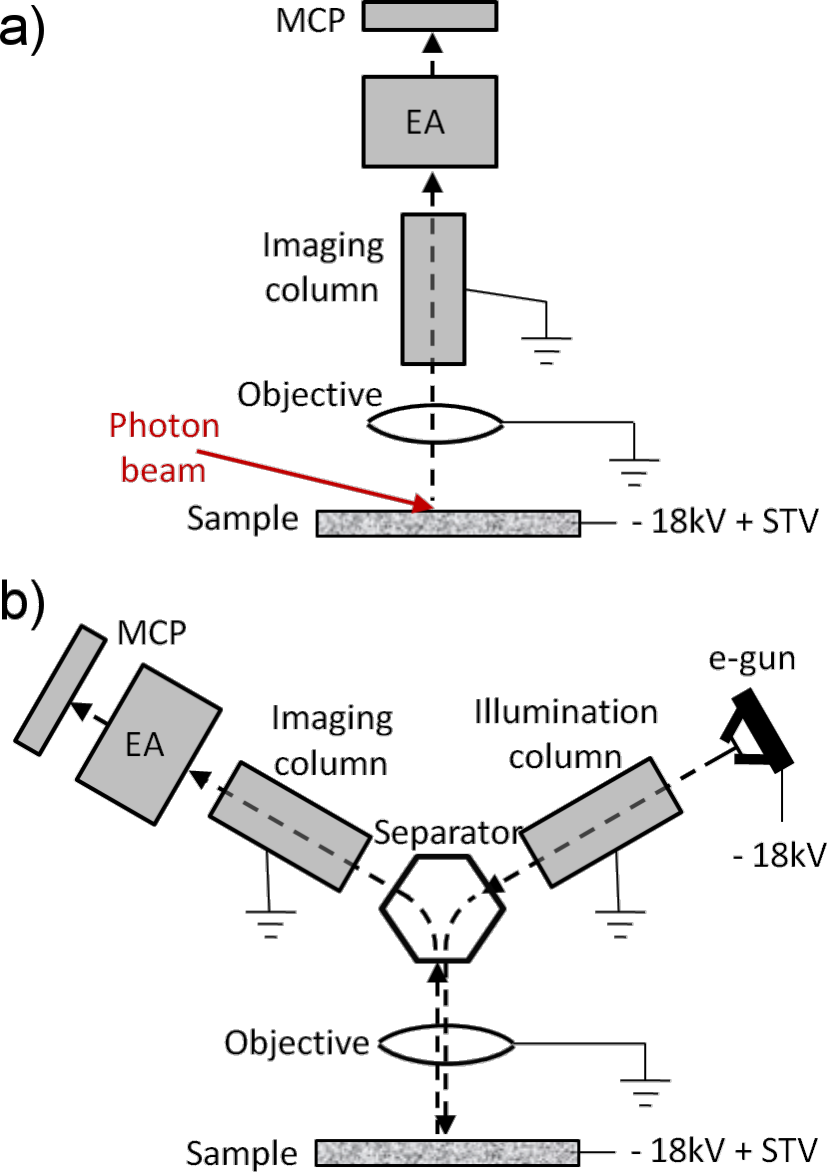
The simplified schematic description of a) XPEEM, b) LEEM. The energy analyzer (EA) is optional in both cases. Panel (b) with the energy analyzer represents also the SPELEEM setup.

Low energy electron microscopy (LEEM) is a surface-sensitive method based on the elastic backscattering of low energy electrons [6,11]. The concept was put forth by Ernst Bauer in the 1960s, and the first operating instrument was demonstrated by Telieps and Bauer [12]. “Low energy” stands for electron energies from a few to several hundred electronvolts. Importantly, due to the high elastic backscattering cross section in the very low energy range (2–20 eV), exposure times are short and data collection becomes possible at video frame rate in most cases. Figure 1a and Figure 1b provide a schematic diagram of typical LEEM and PEEM instrumentation. Backscattered electrons are collected by the objective lens (also known as cathode lens or immersion lens), of which the sample is part. The objective lens, which is the most important optical element of the microscope, accelerates the e-beam to an energy of several keV. The outgoing beam is manipulated by a dedicated set of electron optical elements in the imaging column, which produces a magnified image of the sample. In order to combine the low energy scattering and the high energy imaging stages, a high voltage bias between the sample and the objective lens acts as a decelerating/accelerating potential for the incident/scattered electrons. Besides the electron energy, the backscattering geometry at normal incidence distinguishes LEEM from other more conventional electron microscopies. This necessitates a beam separator, which is used to separate the incident and the scattered electron beams [6].

**Figure 2:**
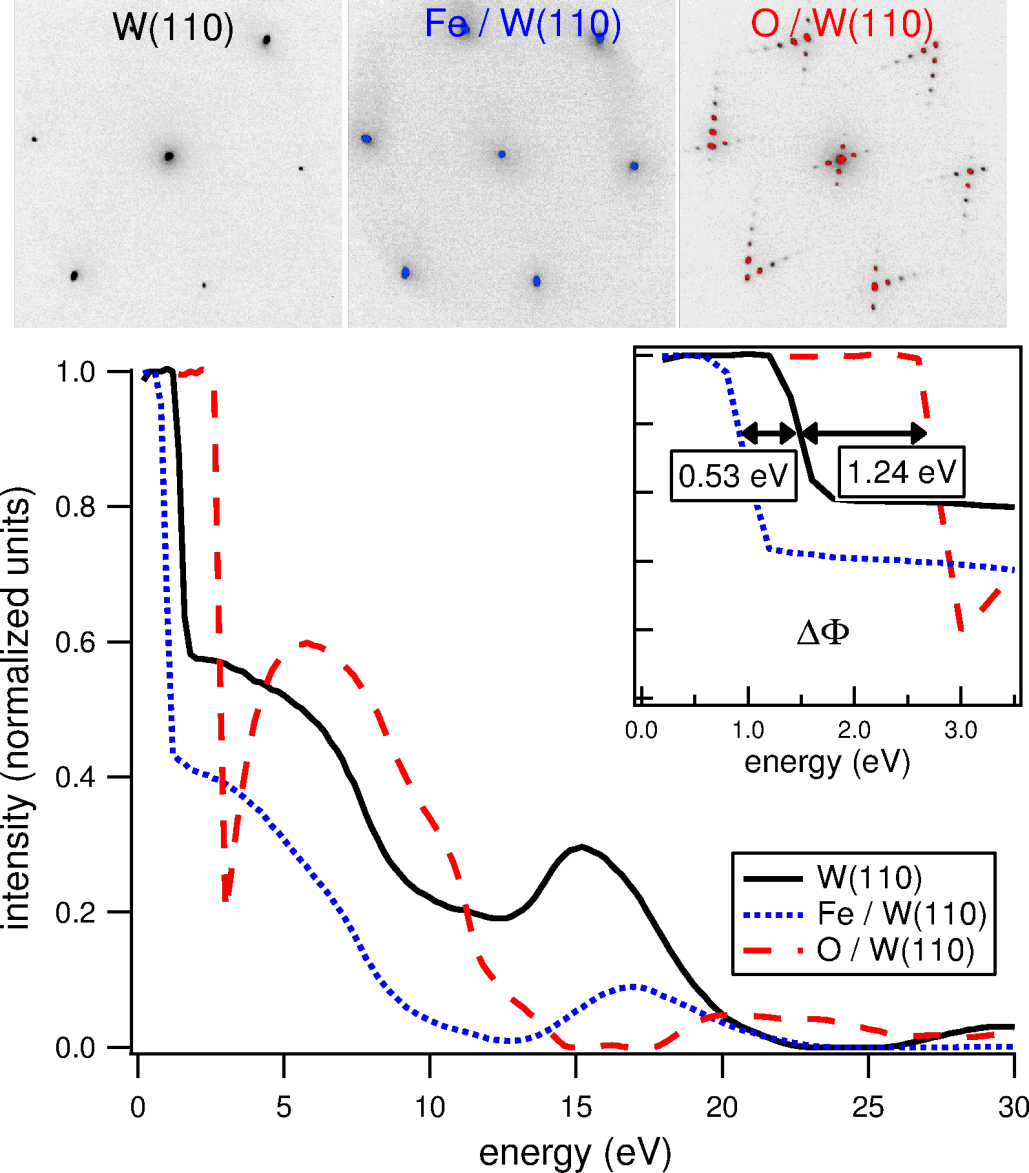
Energy dependence of the (00) beam intensity for clean, Fe-covered and O-covered W(110) surfaces. The top panels show the respective LEED patterns. The inset is a blowup of the MEM–LEEM transition at low energy. The increase (decrease) in the work function due to the presence of O or Fe is seen in the shift of the transition energy.

**Contrast mechanism.** Among all contrast mechanisms available in LEEM, “diffraction contrast” is the one that is most commonly used. This is derived from the strong energy dependence of electron diffraction intensities, making LEEM suitable for studying crystalline systems [13]. The backscattering intensity varies depending on the material, presence of adsorbates, formation of surface reconstructions and other ordered structures, giving the means to distinguish lateral variations in such properties. In the basic operation mode, only one of the low energy electron diffraction (LEED) beams is used to produce an image, in which the energy-dependent intensity provides information about the local morphology and crystal structure. This is done by filtering out undesired diffraction beams in the backfocal plane of the objective lens by using an aperture (usually called contrast aperture). The selection of the specular beam (zero-order diffraction) is commonly referred to as the bright field mode. An illustration of the intensity variations resulting from diffraction contrast is shown in Figure 2. The three curves belong to clean W(110), to W(110) covered with a pseudomorphic Fe monolayer, and to O(1 × 12)/W(110). As seen in the top panels, the first two surfaces give the same (1 × 1) LEED pattern, whereas the oxygen-covered surface features an additional superstructure. Nevertheless, all LEEM *I*(*V*) curves show distinct differences. Similar differences are observed on surfaces with different structure and composition, which produces a contrast between regions of laterally-varying morphology by appropriate choice of the electron energy.

The sharp change in intensity at very low energy, seen in the inset of Figure 2, corresponds to the transition into the so-called mirror electron microscopy (MEM) regime. This MEM–LEEM transition marks the onset for the total reflection of incident electrons as the electron energy is lowered. The threshold energy predominantly depends on the surface work function and on the angle of incidence of the electrons. Therefore, imaging at or near the MEM transition allows to map the local work function as well as the variations in the surface topography. The effect of the work function is clear in the inset of Figure 2, in which the adsorption of oxygen on W(110) results in a work function more than 1.2 eV higher than that of the clean surface, with a corresponding shift in the MEM–LEEM transition. Fe adsorption, instead, induces a less pronounced shift towards a lower work function.

The diffraction contrast is also exploited in the dark field mode, obtained by imaging with higher order LEED beams. The diffraction order is selected by placing the contrast aperture on the desired beam. The resulting real space image gives a direct map of the corresponding structure. No intensity is seen elsewhere, except that originating from the diffuse background of the primary diffracted beam. The lateral resolution is comparable to that of the bright field mode, and the acquisition times, although slightly longer than the bright field operation, can be a few seconds to minutes depending on the intensity in the selected diffraction order.

Due to the short inelastic mean free path (IMFP) at low electron energies below a few hundred electronvolts [14], LEEM is a surface sensitive technique, which probes only a few atomic layers near the surface. Nevertheless, below 10 eV the IMFP considerably increases (up to a few nanometers) giving depth information to LEEM. This is best reflected in the quantum size oscillations in electron reflectivity for thin films on crystalline surfaces [11,15]. As regularly observed in LEEM, the period of intensity oscillations as a function of the electron energy is highly dependent on the film thickness. Beyond their period, the amplitude of these quantum size oscillations depends on the film thickness and the IMFP, which was recently used to extract the IMFP in metal films at very low electron energies [16].

**μ-LEED** operation mode is a natural extension of LEEM. For crystalline surfaces, the backfocal plane of the objective lens contains the diffraction pattern from the probed area, which can be transferred to the detector with the proper lens excitation in the imaging column. By placing a small aperture in the illumination column or at the image plane of the objective lens, the probed area can be limited to a micrometer-sized region, thus giving rise to the micro-probe operation. When the length scale of the structural heterogeneity is below the size of the micro-probe, the contribution of different domains to the LEED pattern can still be sorted out by using LEED in combination with dark-field LEEM imaging. The micro-probe capability is especially useful in quantitative structure analyses of LEED *I*(*V*) curves acquired from single domains on a heterogeneous surface. The first example of a full surface structural analysis at the micrometer scale by using LEED *I*(*V*) in a LEEM instrument was given only recently for the case of the (4 × 4) reconstruction of oxygen on Ag(111) [17].

Beyond the laterally-resolved electron diffraction, LEED measurements in a LEEM instrument have practical advantages such as electron-energy independent spot positions and constant electron flux. The former is due to the acceleration stage at the objective lens, after which the electrons reach 18 keV regardless of the start energy (i.e., the energy of the elastically-scattered electrons at the sample surface). It should be underlined that this is particularly useful in the analysis of energy-dependent *I*(*V*) data.

### X-ray photoemission electron microscopy

PEEM uses UV or soft X-ray photons to stimulate the emission of photoelectrons to probe the state of the emitter. A simplified sketch of an XPEEM setup is given in Figure 1a. Similar to LEEM, it is based on the cathode lens, which accelerates the photoelectrons to an energy of several keV and directs them towards the imaging column of the instrument. The low photon energy of the conventional photon sources readily available in most laboratories presents a limitation, as the information on surface chemistry is available in core-level electronic transitions, which are only accessible by using higher photon energies from few tens of electronvolts to above 1 keV. By providing tunable high-brightness X-ray beams, synchrotron sources greatly extend the application field of XPEEM instruments, which can achieve chemical, magnetic and electronic structure contrast through the implementation of the most popular photoelectron spectroscopies such as X-ray absorption spectroscopy (XAS), photoelectron spectroscopy (XPS), and angle-resolved photoemission spectroscopy (ARPES) [5].

**Figure 3:**
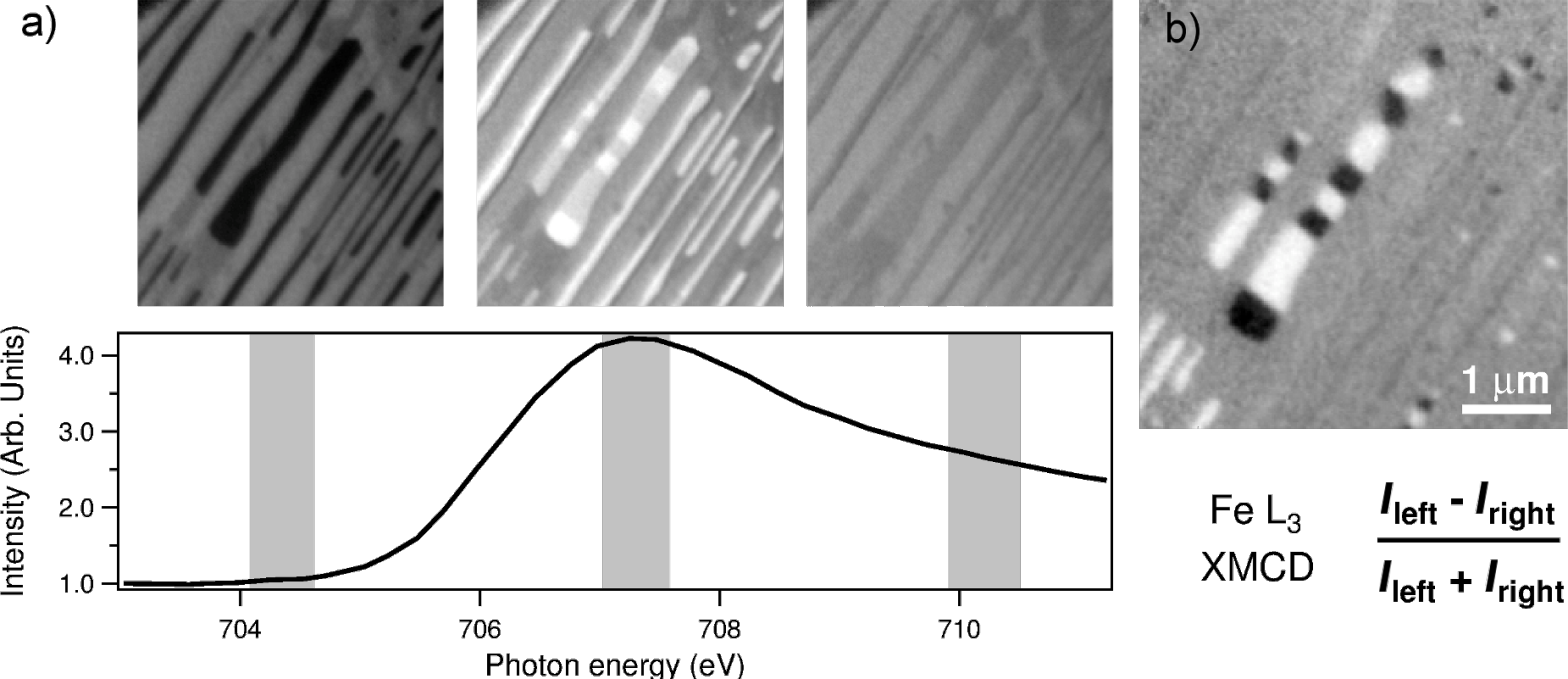
a) Illustration of imaging spectroscopy in XAS mode. Fe nanowires on W(110) appear dark on the left panel at a photon energy of 704.5 eV. At the Fe L_3_ threshold, the wires become much brighter (middle panel). The XAS spectrum below is extracted from the largest nanowire in the center. b) Illustration of XMCD-PEEM imaging. The photon energy is tuned to the L_3_ maximum. The field of view is 5 μm. The start voltage is 3 eV in order to collect secondary electrons. Within the image plane, the X-ray direction is perpendicular to the nanowire axis.

**XAS based methods.** XAS is the only method readily available when using the basic XPEEM instrument installed at a synchrotron beamline with a monochromator in place. Among the variety of detection methods to measure X-ray absorption [18], the secondary photoelectrons are collected in XPEEM as a close approximation to the total photoelectron yield measurement. The local XAS spectra are obtained by acquiring image sequences as a function of the photon energy, which can then be processed in order to extract the intensity variation within any region of interest in the image. Figure 3a illustrates XAS-PEEM imaging spectroscopy on a nanostructured Fe film on W(110). The off-resonant image contrast (leftmost panel) is due to the different secondary photoelectron yield from different surface structures, dominated by the variations in the work function. When the photon energy is tuned to the Fe absorption threshold (middle panel), the elongated Fe nanowires become much brighter, whereas the regions in between barely change intensity. The spectrum seen in the plot in Figure 3a is extracted from an individual nanowire.

**Magnetic imaging.** X-ray magnetic circular and linear dichroism techniques applied to magnetic surfaces constitute major branches of XPEEM research at synchrotrons. Aside from the photon energy, undulator sources provide also the possibility to manipulate the X-ray polarization. The scattering of circularly polarized X-rays is known to carry a contribution from magnetization, which is greatly enhanced at energies corresponding to certain absorption thresholds [19]. Therefore, XPEEM images can be used to obtain the magnetization distribution on a magnetic surface by a simple polarization analysis [20].

The XMCD-PEEM imaging is illustrated in Figure 3b, in which the magnetization distribution of Fe nanowires on W(110) is mapped along the beam direction corresponding to the 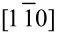 substrate axis. The photon energy is tuned to the Fe L_3_ absorption threshold maximum at about 707.5 eV, and the XMCD image is obtained by taking the difference of the two images with opposing circular polarizations. The wires with magnetization along the perpendicular direction, [001] axis, appear gray as they do not produce an XMCD signal. The black and white regions evident in the central wires are the dipolar domains with magnetization parallel and antiparallel to the beam direction, respectively. Note that the strong magnetic contrast in the case of Fe nanowires is visible even in the single XAS image acquired with circular polarization as seen in Figure 3a (middle panel).

**Core and valence band spectroscopy.** Contrary to XAS-based PEEM, X-ray photoelectron spectroscopy requires a filtering of the kinetic energy of the photoemitted electrons [21]. Therefore, in order to implement XPS in an XPEEM, an energy analyzer has to be installed in the imaging column of the microscope. The most advanced PEEM experiments show a duality between imaging and diffraction operations. The real space image gives a map of the photoelectron intensity, whereas at the backfocal plane the angular distribution of the photoelectrons are imaged. The latter gives access to photoelectron diffraction or angle-resolved photoemission from a micrometer-sized region selected by a field-limiting aperture, which we will refer to as μ-ARPES.

**Dark-field PEEM** is the analogue of the dark-field method in LEEM, such that the same contrast aperture (i.e., diffraction-plane aperture) is used to filter out everything except the emission along a given angle. From a practical standpoint, the main difference of the dark-field XPEEM operation is the necessity to change the sample tilt angle in order to get the diffraction feature through the aperture [22–23]. The angular resolution in the dark-field XPEEM is determined by the size of the contrast aperture, which is typically a fraction of an inverse Å. The lateral resolution is comparable to that of the normal XPEEM operation, well below the micrometer scale. Therefore, dark-field XPEEM makes it possible to probe the electronic structure of small features, which cannot be distinguished in the μ-ARPES mode.

### The SPELEEM at Elettra

Although LEEM and PEEM are widespread, only few instruments that combine both methods can be found in synchrotrons. Some prominent ones are situated at ALBA (Spain), BESSY (Germany), Diamond (UK), MAXLAB (Sweden), NSLS (USA), SOLEIL (France) and SPRING-8 (Japan). Among these, the end station of the Nanospectroscopy beamline at Elettra, the 3rd generation storage ring in Trieste, hosts a spectroscopic photoemission and low energy electron microscope (SPELEEM) [24]. This microscope is the commercial evolution (Elmitec GmbH, SPELEEM III) of the first prototype LEEM with a 120° separator and an energy analyzer, which has pioneered cathode lens spectromicroscopy measurements at synchrotrons during the mid-1990’s [25]. The SPELEEM combines LEEM and XPEEM with energy filter in the same setup: LEEM operation uses an LaB_6_ electron gun and dedicated illumination optics with three condenser lenses, which can deliver a well-collimated e-beam on the sample. In XPEEM operation, instead, the sample is illuminated by the monochromatized X-ray beam produced by the insertion device in the synchrotron ring.

**Figure 4:**
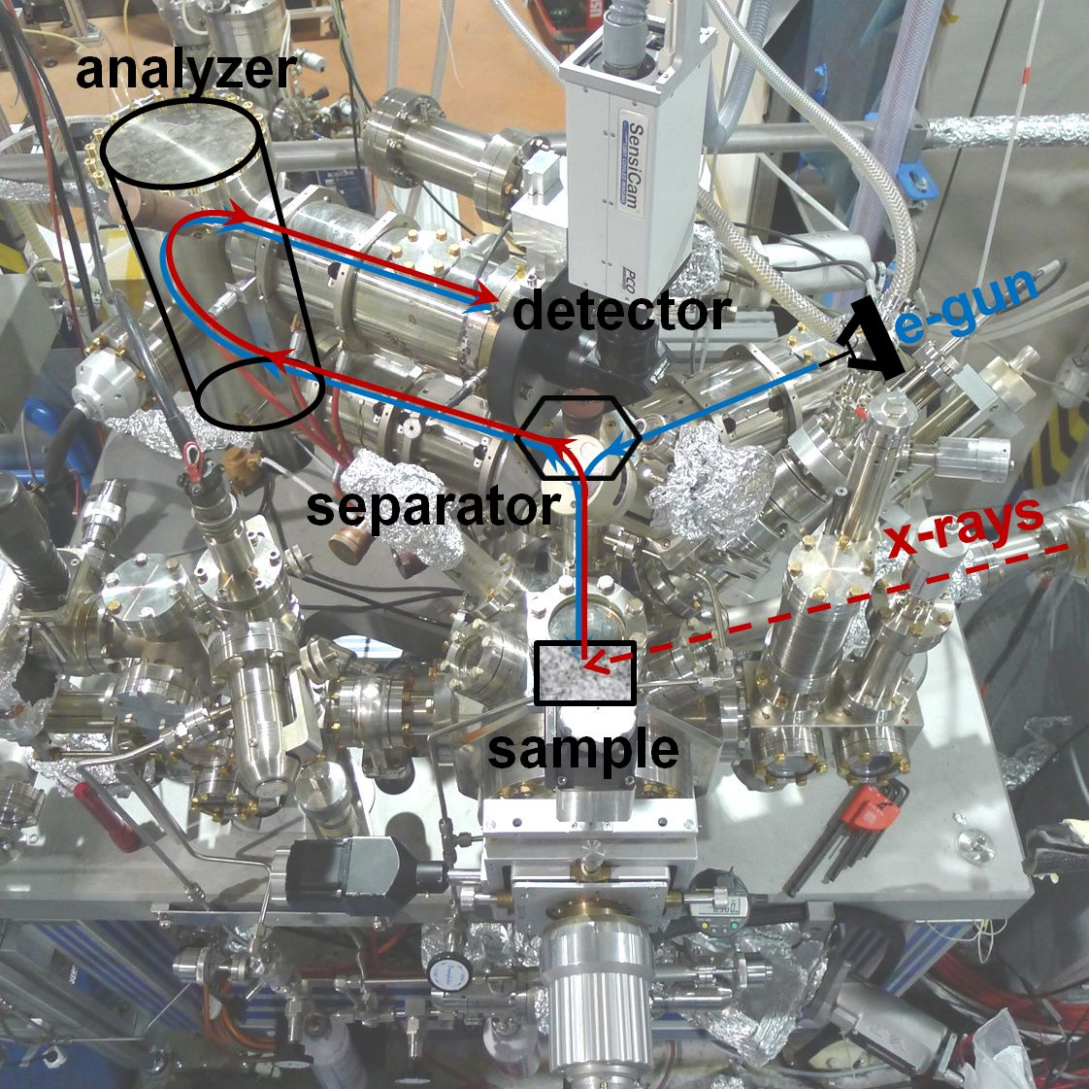
The SPELEEM instrument at the Nanospectroscopy beamline, Elettra Sincrotrone, Trieste. The sketch of the basic setup is superimposed onto the photograph. X-rays arrive from the right at 16° grazing angle to the sample surface.

A photograph of the experimental apparatus is shown in Figure 4. Traces indicating the optical path of the incident and scattered beams in LEEM and XPEEM modes are superimposed onto the photograph. Labels indicate the main electron-optical elements and other essential components. The X-ray beam, traced out as a (red) dashed line entering from the right hand side, is incident on the sample at a 16° grazing angle from the surface plane. The backscattered/emitted electrons are accelerated to 18 keV towards the electromagnetic objective lens. Next element along the optical path is the beam separator, which deflects the e-beam towards the imaging column in which a magnified image of the sample is produced. Depending on the lens excitations, the diffraction pattern at the objective lens backfocal plane can be imaged. At the entrance of the energy analyzer, the electrons are decelerated from 18 keV to 908 eV, the pass energy of the filter. Upon exit from the analyzer, the e-beam is re-accelerated to 18 keV. The final image is projected onto the detector, a chevron multichannel plate followed by a phosphorous screen. The light produced in the phosphorous screen is collected by a CCD camera (PCO Sensicam QE) equipped with an external fan for vibrationless cooling. A more detailed explanation of the SPELEEM optics can be found in reference [25].

**Electron source.** The SPELEEM is equipped with an LaB_6_ thermionic emission cathode (Kimball Physics, model ES-423E, style 06-60). The emitter is a single crystal cut to a cone angle of 60° exposing a 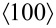 microfacet of 6 μm diameter as tip, which offers high electron flux at a relatively low temperature of the emitter. In the SPELEEM instrument, the electron beam has a flux density of up to 1 × 10^16^ *e*^−^/cm^2^·sec at the surface. The illumination optics focus the beam on the sample to a slightly elliptical shape with diameter of about 90 μm.

**Figure 5:**
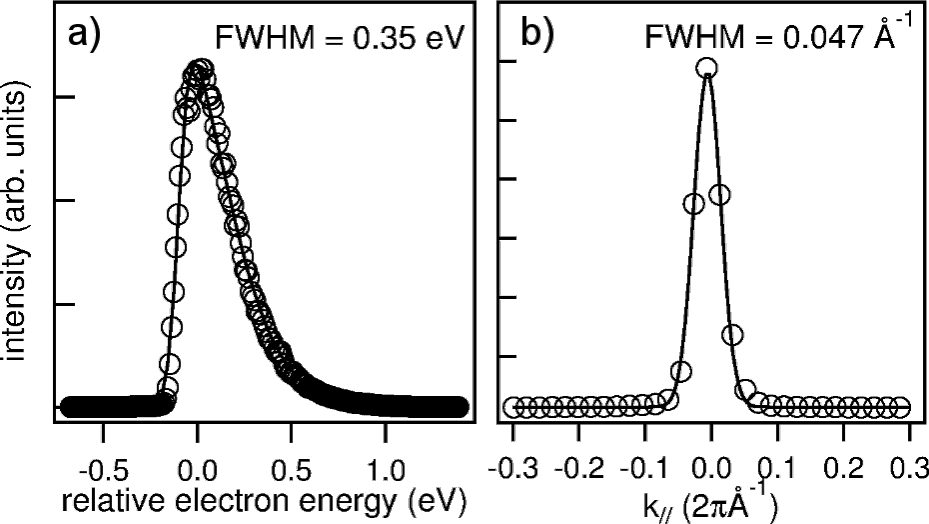
a) The energy distribution of the electron beam emitted from the LaB_6_ source acquired by keeping the sample below the MEM transition using a negative start voltage bias. The intensity-vs-energy curve is obtained in dispersive plane operation, in which the exit plane of the energy analyzer is projected onto the detector. b) The (00) LEED spot profile from W(110).

The energy spread of the LaB_6_ source is set by its operation temperature, reaching 1900 K at a current of 2.12 A. Figure 5a shows the energy distribution of the electron source at the SPELEEM instrument for an operation current of 1.75 A. In order the determine the emitter characteristics, we fitted the experimental data modeling the energy dependence with a simple function taking into account instrumental and thermal broadening [26]. The long tail in the energy dependence of the intensity seen in Figure 5a reflects the LaB_6_ temperature. By fitting the curve with a Fermi function, we determine a tip temperature of about 1750 K. The sharp rise on the left hand side represents the effect of the instrumental broadening. The broadening obtained from this leading edge is about 65 meV, providing a best estimate for the energy resolution of our electron energy analyzer in LEEM operation. Note that this figure is not limited by the size of the contrast aperture, as the angular spread in LEEM is smaller than the aperture size.

The transfer width of the system was measured from the profile of the (00) specular diffraction spot originating from a virtually step-free region of a W(110) crystal, as shown in Figure 5b. Under normal operating conditions, when using a 2 μm illumination aperture and 0.05 μA total emission from the e-gun, the full width half maximum of the Gaussian spot profile was found to be 0.047 Å^−1^. This corresponds to a transfer width of more than 130 Å in real space.

**X-ray source.** Two Apple-II type undulators provide an intense X-ray beam with linear horizontal, linear vertical, or circular polarization from below 10 eV up to 1000 eV [24,27–28]. The monochromator makes use of three gratings to cover the entire photon energy range. A spherical grating is used at the low energies below 50 eV, whereas two variable line spacing gratings of 200 lines/mm and 400 lines/mm cover the ranges of 50–250 eV and 250–1000 eV, respectively. The footprint of the X-ray beam is about 20 × 5 μm^2^ (*H* × *V*), horizontally elongated because of the grazing incidence. A larger area (up to 30 μm) can be illuminated by slightly defocusing the photon beam and by moving it along vertically during image acquisition, although this method usually causes striations in the illumination. The maximum flux is obtained at about 150 eV photon energy and is about 1.8 × 10^13^ photons/s with the exit slit of the monochromator set to 10 μm and with 200 mA synchrotron ring current [29].

**Energy resolution in XPEEM.** The photon source and the SPELEEM operation mode together determine the value of the energy resolution. The resolving power of the VLS400 grating of the beamline monochromator, *E*/Δ*E*, is about 3000 for the photon energy range from 700 to 1000 eV. This figure nearly doubles at lower photon energies [30]. Therefore, in the majority of practical cases, the microscope energy analyzer is the limiting factor in terms of energy resolution.

In energy-filtered imaging and diffraction, where images are collected at a well-defined photoelectron kinetic energy, the energy resolution is mainly determined by the size of the analyzer exit slit. Two slits of different width can be used to set the bandpass of the energy filter, corresponding to energy windows of 0.33 eV and 0.79 eV, respectively. Note that the best energy resolution from the instrument can be obtained in the *μ*-spectroscopy operation, in which the dispersive plane of the analyzer is imaged onto the detector. In this way, the resolution is mainly determined by the radius of the analyzer hemisphere (*r*_0_ = 10 cm), the pass energy (*E* = 908 eV), and the angular spread before the analyzer (*α* = 5 mrad full width) given by the size of the contrast aperture (*d* = 20 μm, smallest aperture), which also acts as the entrance slit. The energy spread Δ*E* (full width at half maximum) is parameterized as [31]:

[1]
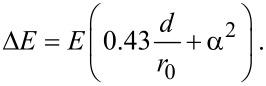


**Figure 6:**
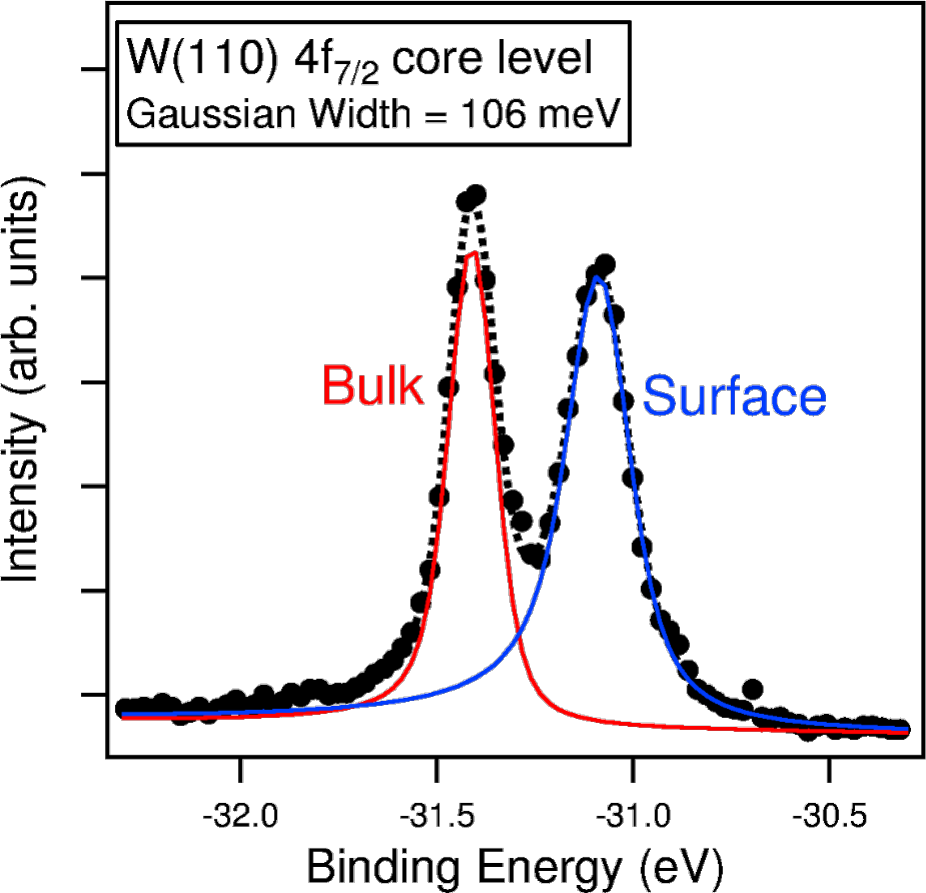
Tungsten 4f_7/2_ core level spectrum from a clean W(110) surface acquired in dispersive plane mode. The photon energy is 90 eV. The acquisition time is 80 s. The Lorentzian broadening for the bulk peak was fixed at 60 meV, with an asymmetry parameter of 0.035. The contrast aperture, which acts as the analyzer entrance slit, is 20 μm.

Inserting the values listed above, the best resolution is estimated to be 101 meV. The experimental energy resolution of the SPELEEM was measured from the W 4f core level of the clean W(110) surface. The dispersive plane spectrum and the corresponding Doniach–Šunjić fit are displayed in Figure 6 [32]. The resulting full-width half-maximum of the Gaussian broadening is found to be less than 110 meV for the optimal conditions (smallest contrast aperture, low photon flux, small field-limiting aperture), which is in excellent agreement with the above estimation. For the usual operating conditions with less stringent parameters, the energy resolution was found to be about 150 meV.

**Figure 7:**
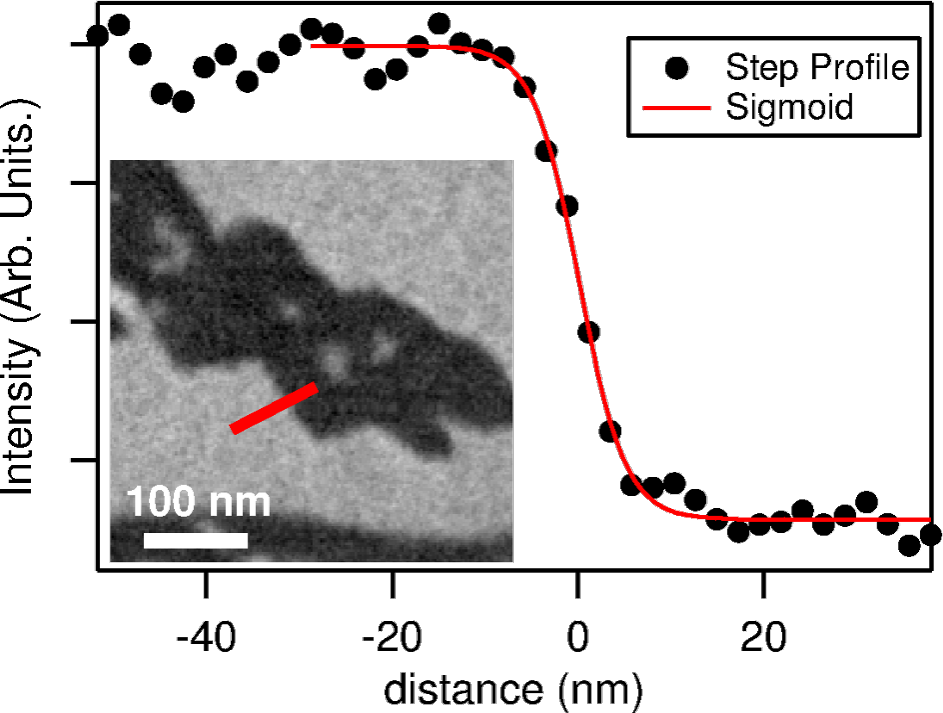
Lateral resolution in LEEM. The inset shows a Ni monolayer island (dark) on W(110). The profile in the plot is marked on the image. The full width of the sigmoid function is 8.2 nm. Averaging over several profiles, the value is found to be 8.6 ± 1.2 nm.

**Lateral resolution.** The SPELEEM spatial resolution is mainly determined by the spherical and chromatic aberrations of the objective lens. LEEM performs better compared to XPEEM. Low energy electron diffraction beams are generally much sharper than the broad photoelectron emission angles. As a result, the angular spread (thus spherical aberrations) in LEEM is considerably reduced compared to XPEEM. The same is true also for the energy spread and the chromatic aberrations, which are again reduced in the case of LEEM. The lateral resolution in LEEM mode for the SPELEEM at Nanospectroscopy is demonstrated in Figure 7. The plot shows the variation of the LEEM intensity across a profile through a ML thick Ni island on W(110), along with a sigmoid fit. The width of the sigmoid represents the instrumental broadening, corresponding to the distance identified by using the 16–84% intensity variation criterion often used to characterize the lateral resolution. By averaging over several profiles across the image, we obtained a value of about 9 nm. The same value is obtained when measuring the width of steps (providing phase instead of amplitude contrast) on clean W(110). Note that this value is better by more than 20% compared to the performance prior to the installation of the new electron source. This improvement is based on the smaller energy spread and the reduced transfer width, which are reported in Figure 5. The lateral resolution may possibly be further reduced by using a field-emission source with superior characteristics.

The spatial resolution in XPEEM is about 30 nm [24]. For comparison, consider that the wide angular spread observed when imaging the inelastically scattered energy-loss electrons in LEEM gives a spatial resolution of about 18 nm, similar to XPEEM [33]. It is important to note that the lateral resolution in XPEEM suffers from space-charge effects due to the highly brilliant synchrotron pulses [29]. The electron–electron interaction within the photoelectron pulse produced by the X-ray pulse (which has a low duty cycle of about 1/50) results in the degradation of the image quality as well as increasing the energy spread. As a result, even the aberration-corrected instruments are limited to a moderate lateral resolution in XPEEM [34]. Similar effects were not observed in LEEM with its monochromatic LaB_6_ cathode providing a much lower current density than the peak photoelectron current in XPEEM.

### SPELEEM studies of graphene epilayers

LEEM has found ample use in graphene research with its high structural sensitivity and video acquisition rate allowing for dynamic measurements of film growth. In such experiments, graphene is typically obtained by the chemical vapor deposition (CVD) technique. CVD utilizes transition metal catalysts as a means to promote the dissociative adsorption of gases such as ethylene or methane, which can readily deliver the carbon atoms required for island nucleation and growth. LEEM is widely employed to image the growth process; the accessible parameter space is explored by varying the gas pressure and/or sample temperature in a manner compatible with the operation limits of the instrument. In this regard, LEEM permits operation at elevated sample temperatures (approximately up to 1000°C) by using the experimental chamber as a gas flow reactor up to pressures approaching 1 × 10^−5^ mbar, still ensuring a lateral resolution nearing 10 nm. The growth of graphene on a variety of transition metal substrates provide catching examples of the potential of the LEEM method [35–37]. For instance, by measuring subtle variations in the low energy electron reflectivity of the substrate upon ethylene uptake, it has been shown that LEEM can quantitatively monitor the time evolution of the C adatom lattice gas that anticipates island nucleation. Tiny variations of the carbon coverage were detected in these experiments, achieving sensitivities below 0.1% of a ML. These experiments provided a formidable means to access to the thermodynamics governing carbon segregation, graphene nucleation and film growth [36–38]. In other cases, LEEM imaging was used to monitor the intercalation of adspecies below graphene [39–41]. As a further advantage, LEEM allows for thickness determination in multilayer systems, through the exploitation of quantum size contrast. Nevertheless, the understanding of the low energy electron reflectivity is not always straightforward, since it is extremely sensitive to the substrate–film interaction, as has been recently demonstrated by an ab initio study that clarified the interpretation of LEEM-*I*(*V*) curves [42].

An important aspect is the integration of LEEM with low energy electron diffraction (LEED). Diffraction experiments permit a full characterization of the crystal structure and quality of graphene. For instance, μ-LEED methods have been devised to quantify the short-range roughness of multi-thickness SiO_2_-supported and suspended exfoliated graphene films [43]. To date, the most frequent application of LEEM/LEED has been the study of rotational domains and complex moiré patterns in a wide variety of graphene epilayers. A notable example is that of graphene on Re(0001), in which a moiré cell made out of (10 × 10) graphene unit cells over (9 × 9) Re unit cells was determined in a μ-LEED experiment performed in a SPELEEM microscope; the atomic positions in the unit cell were subsequently obtained by means of ab initio calculations, which could prove the very large corrugation of graphene in this system and establish a correlation between C 1s binding energy and C–substrate separation [44]. On the same system, μ-probe diffraction analyses were carried out in combination with dark-field imaging, investigating the carburisation of the Re(0001) substrate as a function of temperature [45]. The literature shows a plethora of other experiments exploiting the LEED capabilities of LEEM with notable examples of graphene on single and polycrystalline copper [46–48], nickel [49] and on non-threefold crystalline substrates such as Ir(100) [50–51] and Fe(110) [52].

There is also a growing literature on XPEEM applications in graphene research. In particular, μ-ARPES available in the SPELEEM has been successfully applied to exfoliated [53–54] as well as epitaxial graphene grown on metal [48,55–56] and SiC substrates [57]. In many studies μ-ARPES was employed to access the π-band of graphene, to quantify the doping in graphene and to verify the strength of the film–substrate interaction. At length scales below the resolution of the μ-probe approach, the recently introduced dark-field XPEEM method is ideally suited to compare the density of states (DOS) of different, adjacent types of graphene exhibiting distinct electronic structure properties. Laterally resolved XAS was also utilized in an isolated study on exfoliated graphene, in which selected features in the K-edge spectrum of C were studied as a function of the thickness of graphene. A splitting of the π^*^ resonance was observed in multilayers and ascribed to specific interlayer states [58].

**The intercalation of Au below graphene on Ir(100).** Recently investigated by using a wide range of microscopy methods and theory, the graphene/Ir(100) system exhibits unique morphological and electronic properties, which originate from the different film and substrate symmetries [50]. At temperatures above 800 °C, micrometer-sized single layer graphene crystals can be obtained upon exposure to ethylene, oriented at 3° with respect to the main substrate direction. By cooling the sample from growth to room temperature, a phase transformation occurs in the graphene film, which develops neighboring phases characterized by flat and buckled morphology. Adjacent striped-shaped domains of different carbon surface density alternate on the film at microscopic length-scales, relieving the strain accumulated upon cooling to room temperature. Most interestingly, the buckled graphene phase is characterized by large and extremely regular one-dimensional ripples showing a periodicity of 2.1 nm. Dark-field PEEM experiments have demonstrated that the buckled graphene phase shows a negligible DOS at the *K* point of the π-band. These results point to the disruption of the Dirac cones and the formation of a metal-like DOS. Surprisingly, the hybridization of the π-band with Ir states is due to the chemisorption of just 11% of the C atoms in the unit cell of the buckled phase [50].

In order to modify the graphene–substrate interaction in this system, we have intercalated Au at elevated sample temperature, taking advantage of the fast diffusion of Au adatoms under such conditions. Au is expected to exert a weaker interaction than Ir, hindering the formation of the chemisorption bonds such as those observed in the buckled graphene phase on Ir(100). In our work, the related variations in C–substrate bonding and the electronic structure of graphene were quantified in μ-XPS and μ-ARPES experiments, taking the pristine graphene/Ir(100) system as reference. Experimentally, Au was deposited by using an e-beam evaporator (Elmitec GmbH) at a flux of 0.059 eML_Ir(100)_ (equivalent monolayer of Ir(100)) per minute at a sample temperature of about 600 °C.

**Figure 8:**
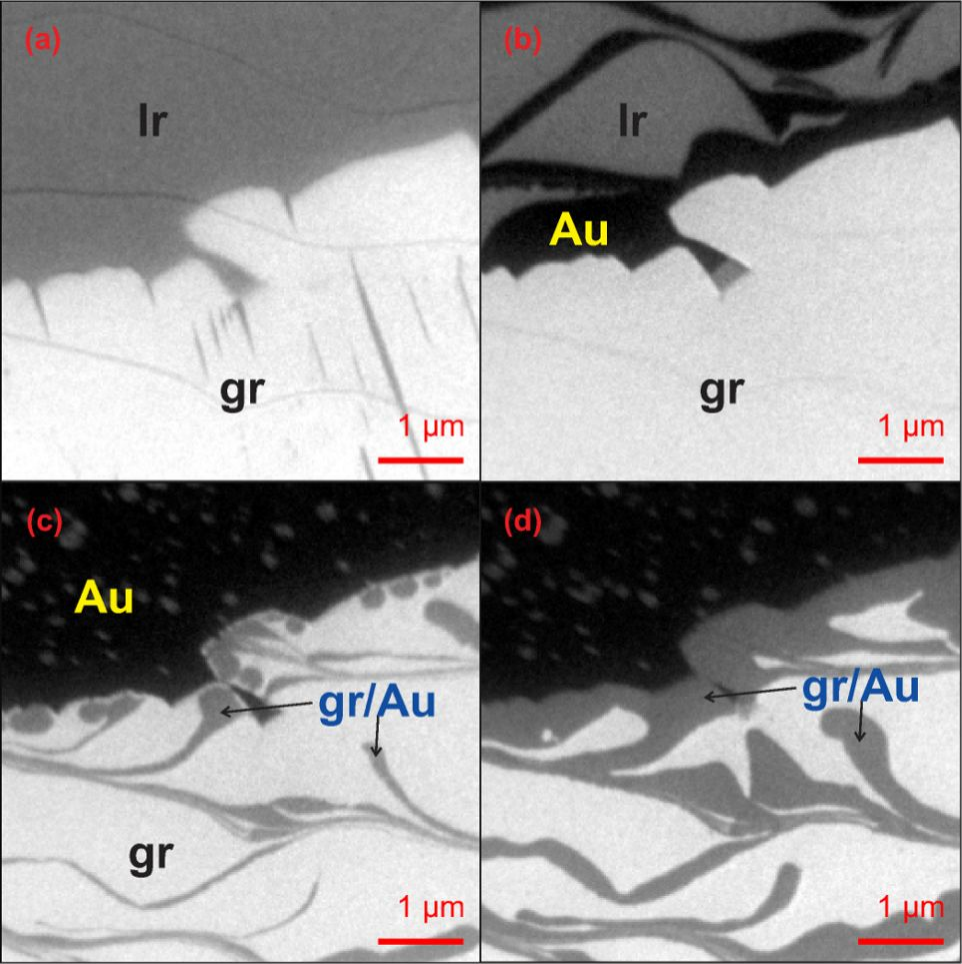
LEEM images at a start voltage of 12 eV illustrating the evolution of the graphene/Ir(100) interface upon deposition of Au. A large graphene crystal (gr) is visible in all images (lower half), brighter than the Ir surrounding it (upper half). (a) Initial configuration of the sample at *T* = 520 °C. (b) The same area after a dose of 0.25 ML Au at sample temperature of 600 °C. Au (dark areas) has decorated steps and step bunches. (c) The same area after a dose of 0.85 ML of Au. At this stage, Au has entirely covered the initially bare Ir surface and the intercalation under graphene has just started (darker areas). Note also that small graphene islands have nucleated on the Au/Ir surface. (d) The same area after a dose of 0.9 ML of Au.

The evolution of the interface upon increasing Au dose was monitored in situ employing LEEM. Figure 8a illustrates the initial state of the surface, with a graphene island (bright) located in the lower half of the image. The same image (upper half) shows the bare Ir surface, rendered in medium gray; a few thin curved lines can be spotted here, which identify morphological features of the surface such as steps and step bunches. The first stages of the Au growth are shown in Figure 8b, the Au-covered areas appearing darker than the Ir substrate at the chosen electron energy (12 eV). As can be seen, Au has already decorated the steps and has formed a step-flow growth front, its local thickness being just one layer. Note that Au is not found on or below graphene, but adsorbs exclusively on the iridium substrate. Only when the bare Ir surface has been fully covered, the intercalation process starts. At this stage, the Au growth front propagates quickly under the film, as shown in Figure 8c and Figure 8d, until a full Au monolayer is formed below graphene.

There are two interesting findings that need to be highlighted. First, it appears that Au can intercalate below graphene only after accumulation of a full Au ML at the island edges, and before the nucleation of second or multilayer Au islands. Second, small graphene islands nucleate at the Au-covered surface far from the large graphene crystal, as was verified by μ-LEED analysis. The former observation gives the order of energetics for Au adsorption on Ir versus its intercalation under graphene. The intercalation is facilitated by the lifting of the chemisorbed graphene edges upon Au adsorption at the edges. To explain the appearance of small graphene islands on Au, we must consider that 0.1 ML carbon is chemisorbed to the Ir surface, forming a carbidic c(2 × 2) structure [51]. In such a structure, C is strongly bound to the substrate, with a binding energy of almost 8 eV at a coverage of 0.5 eML_Ir(100)_ [59]. We note, however, that Au binds strongly to other noble metal substrates. For instance, on Rh(110) the binding energy of Au is 3.5 eV at ML-coverage [60]. Due to the strong interaction with Ir, the adsorption of Au weakens the C–Ir bonds, and the high density of C adatom gas on the Au layer readily condense to form graphene islands.

Upon subsequent cooling to room temperature, the morphology and structure of graphene remain unchanged. In fact, we could not detect any evidence of phase transformation or formation of stripe-shaped domains resembling those of observed on Ir(100). Instead, LEEM imaging at high lateral resolution evidenced the formation of wrinkles in the graphene film, a process which helps relieving the thermal strain, because of the different thermal contraction of film and substrate. Similar features have been previously observed for graphene on Pt(111) and Ir(111) surfaces [61–62]. Importantly, no coincidence structures are observed in the LEED pattern, which exhibits only the first order graphene spots plus an extremely week moiré structure, identical to that observed on the flat phase of graphene on Ir(100). This finding further confirms that, after Au intercalation, graphene is entirely physisorbed and no chemisorption bonds are established between C and the substrate.

The C 1s μ-XPS spectrum of the graphene/Ir(100) system exhibits two components. The dominant one, at about 283.95 eV binding energy, has been previously ascribed to physisorbed C [50], consistent with the binding energy values observed for weakly-interacting graphene on a variety of fcc(111) and hcp(0001) substrates [44,63]; the second peak appears at higher binding energy (about 284.9 eV) and is due to the small fraction of chemisorbed carbon atoms in the buckled phase. Conversely, the C 1s spectrum measured on the graphene/Au system shows only the physisorbed component, proving that no chemisorption to the substrate has been established upon cooling to room temperature.

**Figure 9:**
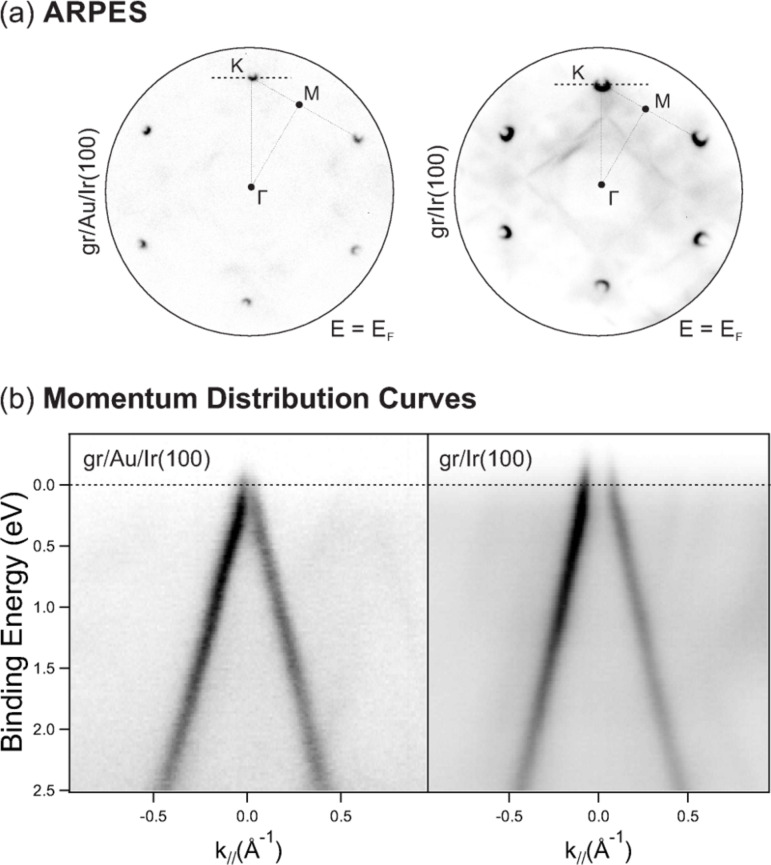
Graphene on Au/Ir(100) (left column) and Ir(100) (right column). (a) μ-ARPES near *E*_F_; the high symmetry points in the first Brillouin zone (FBZ) are indicated. Photon energy is 40 eV. The probed area has a diameter of 2 μm. (b) Momentum distribution curves along the normal to the Γ–K direction, as indicated by the dashed line in (a).

Clearly, the Au layer induces important variations in the graphene–substrate interaction, which in turn affect the charge transfer processes occurring between substrate and film and, consequently, the doping. Figure 9 shows μ-ARPES patterns (a, top) and momentum distribution curves (b, bottom) along the high symmetry directions for the graphene/Au system (on the left-hand side of the Figure) and pristine graphene on Ir(100) (on the right-hand side), respectively. Visual inspection of the full μ-ARPES pattern at *E*_F_ shown in (a) instantly reveals that Au intercalation is manifested by a change in the doping: The circular features identifying the Dirac cones for the graphene/Au system appear narrower than those recorded on pristine graphene, suggesting that the Dirac energy is now closer to the Fermi level. By accurately fitting of momentum distribution curves we could determine a positive doping of just 0.09 ± 0.06 eV for graphene/Au/Ir(100), which has to be compared with the value of 0.42 ± 0.03 eV obtained on graphene/Ir(100) [50]. Our results are in fair agreement with *μ*-ARPES data for the graphene/Au/Ni(111) system [64]. On the Ni substrate, the Au intercalation leads to a non-rigid shift of the bands of graphene towards lower binding energies, the *π*-band moving by approximately 2 eV; the Dirac energy *E*_D_ is found at just 25 meV above the Fermi level, so that quasi free-standing conditions for the film are claimed. Referring to an analytical model [65–66], the Fermi level shift in graphene translates to the graphene–Au distance beng in the range of 3.4–3.6 Å, slightly larger than the calculated graphene–Ir distance [50].

### Self-organized nanomagnets

*S*elf-organization may be ascribed the general meaning “spontaneous appearance of a particular form”. Even though the definition may be stretched about to describe nearly all observed shapes in nature, static and dynamic, we assign the term to the formation of regular structures. SPELEEM methods perfectly lend themselves to studies of self-organization phenomena, particularly in the field of nanomagnetism. In a nutshell, LEEM is used to monitor the growth process in real time at high temperatures; spectromicroscopy with XPEEM provides the chemical map of the resulting heterogeneous surface; and finally XMCD-PEEM reveals the magnetization distribution of this nanostructured surface.

**Stress-induced adsorbate stripes** have been recently observed on crystalline surfaces at high temperatures. The mechanism is based on the competition between the cost of a boundary and the gain due to long range elastic interactions between boundaries [67]. The temperature determines the relative strength of the long- and short-ranged energy terms [68].

**Figure 10:**
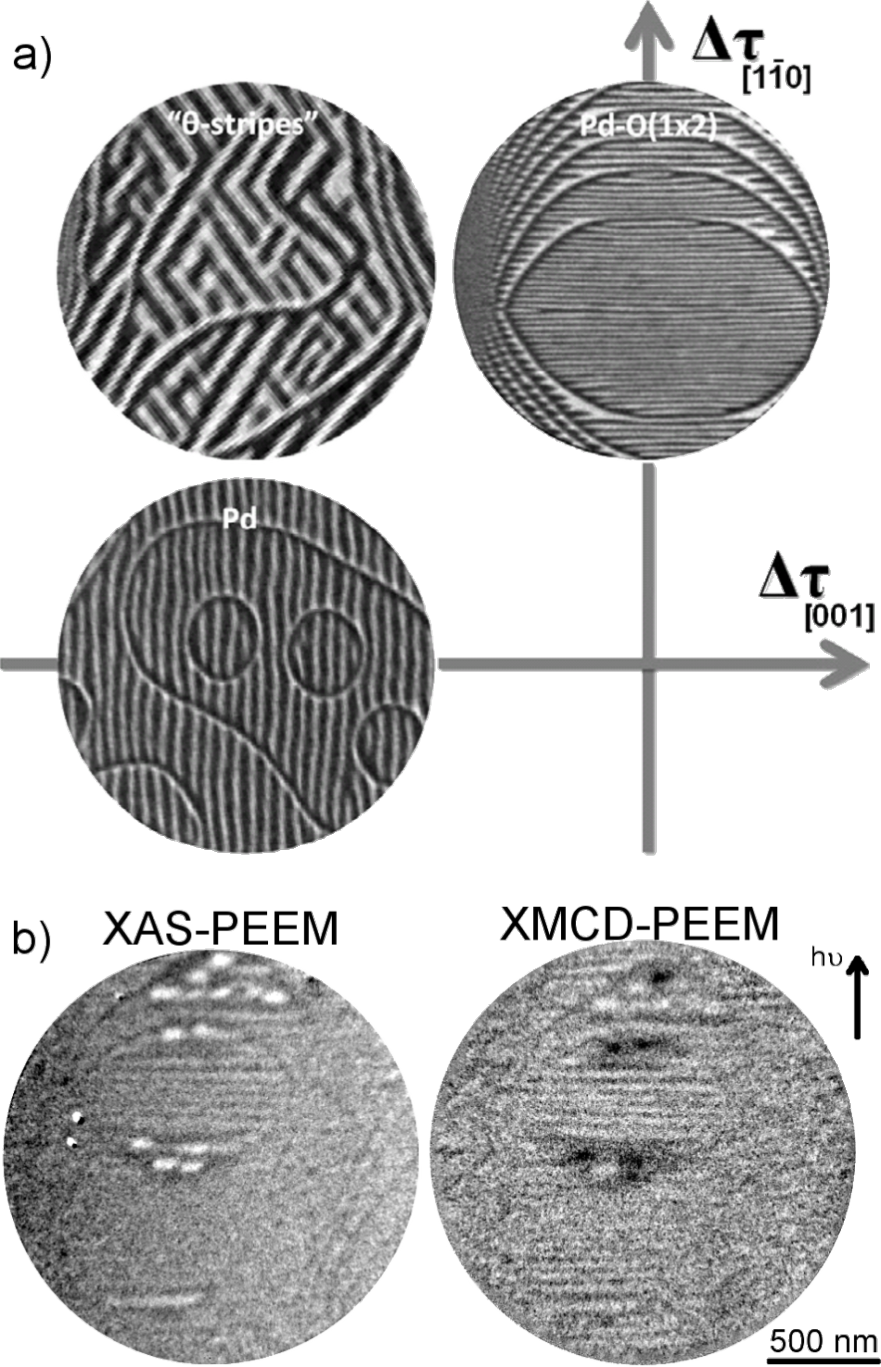
a) LEEM images (2 μm diameter) of monolayer Pd stripes on W(110). The lower left panel shows Pd on a clean substrate, whereas the other two panels display the progressive change in pattern anisotropy upon addition of 0.1 ML and 0.33 ML oxygen. (Reprinted from [69]. Copyright 2011 IOP Publishing.) b) Fe grown on Pd–O stripes at 225 °C. Left panel is the XAS-PEEM image at the Fe L_3_ edge showing the Fe distribution. On the right, the Fe XMCD image indicates that the wires are uniformly magnetized perpendicular to the long axis. (Reprinted from [70]. Copyright 2013 Elsevier.)

In the case of monolayer Pd stripes on W(110) forming at about 1100 K [68], it was recently shown that the addition of oxygen modifies the pattern anisotropy while preserving the periodic structure [69]. Moreover, the Pd–O bispecies layer is stable upon lowering the temperature from above 1000 K down to room temperature. The different adlayer patterns that can be obtained by varying the amount of oxygen on the surface are depicted in Figure 10a. The changes in the pattern anisotropy are driven by the magnitude and sign of stress variations on the surface, which are both dependent on the presence and amount of oxygen [69].

The stability of the Pd–O stripes on W(110) at lower temperatures allows for a further growth of magnetic wires following the self-organized template. Fe/Pd–O stripes on W(110) have been demonstrated very recently [70]. XPEEM imaging at the Fe L_3_ absorption threshold has confirmed the expected Fe distribution as seen in Figure 10b (left panel). In the resulting picture, Fe preferentially wets the Pd-covered parts of the striped template. Furthermore, at about 200 °C Fe and Pd rearrange to make a surface alloy with a Pd-rich surface layer. The magnetization of the FePd stripes was found to be along the 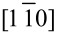 direction perpendicular to the stripe axis, as shown in the XMCD map in Figure 10b (right panel). This is a surprising confirmation of the magnetocrystalline anisotropy strength dominating the shape anisotropy.

**Iron oxides** find wide application in several fields of research, among others magnetism and heterogeneous catalysis. In both cases, the heteroepitaxial growth of nanostructured FeO*_x_* offers a versatile means to tune the material properties. Recently, SPELEEM techniques were applied to characterize the reactive growth of FeO*_x_* on Ru(0001) [71]. Fe growth in an oxygen ambient (5 × 10^−7^ mbar) at 900 K resulted in the formation of perfectly triangular micrometer-sized Fe_3_O_4_ islands on a FeO wetting layer. The combination of spatially-resolved XPS and XAS spectra, along with μ-LEED patterns, allowed the unequivocal identification of the specific iron-oxide phases.

**Figure 11:**
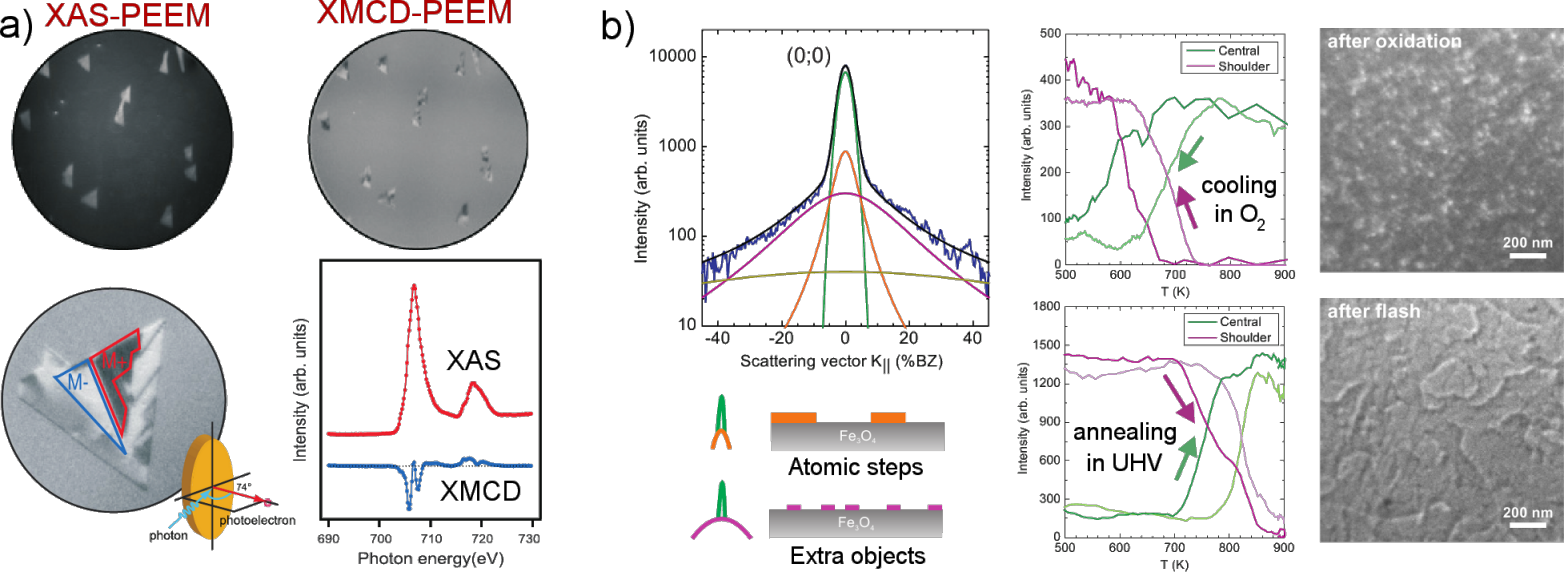
a) Magnetite islands and the FeO wetting layer on Ru(0001). Top panels show the island and magnetization distribution within a region of 30 μm diameter, illuminated homogeneously by vertically scanning the photon beam during acquisition. Bottom panels show the details of the magnetic domains (left, field of view 4 μm) and the Fe L_3_ XMCD spectrum extracted from a single domain (right). (Reprinted with permission from [72]. Copyright 2012 American Physical Society.) b) Morphology of Fe_3_O_4_/Pt(111) from LEEM and μ-LEED spot profile analysis. The large tail in the (00) spot profile (seen on the left) is identified with the formation of oxygen-related agglomorates as sketched below. Middle panels show the effect of cooling/annealing on the spot profile. LEEM images on the right (at an energy of 24 eV) present the surface before and after the annealing cycle. (Reprinted with permission from [73]. Copyright 2012 American Physical Society.)

From the screening of substrate core-level photoelectrons, the thickness of the micrometer-sized magnetite islands was found to be about 1 nm, which corresponds to two unit cells [72]. XMCD-PEEM measurements on these ultrathin islands (seen in Figure 11a) show that magnetite preserves its ferrimagnetic properties at 1 nm thickness up to 520 K (above which the morphology changes irreversibly). This observation corresponds to the thinnest magnetite crystal that shows magnetism.

Beyond the self-organized crystal shapes at the micrometer scale, epitaxial iron-oxide films provide a variety of complex surface reconstructions at the atomic scale as usual for oxide surfaces [74]. Fe_3_O_4_ films on Pt(111) are known to give a (2 × 2) reconstruction with an additional moiré superstructure. Nevertheless, the details of the Fe_3_O_4_ surface structure is still under study. The recent work by using an aberration-corrected XPEEM-LEEM setup, SMART (BESSY II, Helmholtz Zentrum, Berlin), showed distinct differences between LEED *I*(*V*) curves obtained from surfaces with differing preparation pathways [73]. The role of surface preparation was revealed in the LEED spot-profile analysis of the Fe_3_O_4_(111) surface as summarized in Figure 11b, which showed the formation of oxygen-related defects. In particular, the different contributions to the LEED spot-profile were observed in real-time as a function of temperature, which resulted in a model of oxygen-induced extended surface defects [73].

Aside structure and magnetism, transformations between different iron-oxide phases were studied by using the SPELEEM methods. In the above example of FeO*_x_* growth on Ru(0001), further oxidation by using NO_2_ as atomic oxygen source resulted in the transformation of the FeO wetting layer to hematite (α-Fe_2_O_3_) and the triangular Fe_3_O_4_ islands to maghemite (γ-Fe_2_O_3_) [71]. In an independent study, the real-time observation of thermal reduction with LEEM and LEED was crucial in understanding the reversible changes in thin magnetite and hematite films grown on several substrates [75]. In particular, annealing in UHV led to substrate-dependent transformations of the iron oxide thin film: from hematite to magnetite on a Pt(111) substrate and vice-versa on Ag(111). The conversion has been explained as a competition between the dilution of Fe cations in the substrate, predominant only in the former case, and the desorption of oxygen.

## Summary

We have given a review of SPELEEM methods along with recent applications in the fields of graphene and nanomagnetism. The extensive introduction to LEEM and XPEEM techniques illustrates the basic operation principles, with the intention to serve as a guideline for those unfamiliar with the field. A working example of a SPELEEM instrument is the one available at the Nanospectroscopy beamline at Elettra. Details on the instrumental aspects of the Nanospectroscopy microscope have been given both as an update on the performance of this particular setup, and also as a reference for the typical SPELEEM properties regarding parameters such as energy resolution, lateral resolution, electron beam characteristics and the transfer function of the instrument. After the instrumental part, recent scientific activity in graphene research by using LEEM-PEEM methods have been reviewed. Then, the original example of Au intercalation at the graphene–Ir(100) interface has been presented, showing the effective role of Au in breaking the C–Ir chemisorption bonds and in restoring the neutral Dirac point nearly at the Fermi level. The review has been concluded with examples on self-organized nanomagnetism studies taking advantage of the possibility to perform magnetic imaging with an XPEEM, based on the X-ray magnetic circular dichroism contrast.
